# 3D-Printed Insole for Measuring Ground Reaction Force and Center of Pressure During Walking

**DOI:** 10.3390/s25082524

**Published:** 2025-04-17

**Authors:** Le Tung Vu, Joel Bottin-Noonan, Lucy Armitage, Gursel Alici, Manish Sreenivasa

**Affiliations:** School of Mechanical, Materials, Mechatronic and Biomedical Engineering, Faculty of Engineering and Information Sciences, University of Wollongong, Wollongong, NSW 2522, Australia; ltv344@uowmail.edu.au (L.T.V.); joelbn@uow.edu.au (J.B.-N.); alucy@uow.edu.au (L.A.); gursel@uow.edu.au (G.A.)

**Keywords:** biomechanics, kinetics, foot forces, wearable sensors, plantar pressure

## Abstract

Ground reaction force (GRF) and center of pressure (COP) during walking are two important measures that could be used in a range of applications, from the control of devices such as exoskeletons to clinical assessments. Recording these measures requires fixed laboratory equipment such as force plates or expensive portable insoles. We present an alternative approach by developing a 3D-printed insole that uses pneumatic chambers and pressure sensors to estimate the net GRF and the anterior–posterior COP position. The intentionally simple design, using just two pneumatic chambers, can be fabricated using standard 3D printing technology and readily available soft materials. We used experimentally recorded data from a motion capture system along with parameter identification techniques to characterize and validate the insole while walking at different speeds. Our results showed that the insole was capable of withstanding repeated loading during walking—up to 1.2 times the body weight—and possessed a bandwidth high enough to capture gait dynamics. The identified models could estimate the GRF and the anterior–posterior COP position with less than 9% error. These results compare favourably with those of commercially available instrumented insoles and can be obtained at a fraction of their cost. This low-cost yet effective solution could assist in applications where it is important to record gait outside of laboratory conditions, but the cost of commercial solutions is prohibitive.

## 1. Introduction

Assessments of the magnitudes and locations of external loads applied to the lower limbs during locomotion through ground reaction forces (GRFs) and centers of pressure (COPs) are crucial for understanding the biomechanical mechanisms of gait. Understanding these parameters can play a significant role in clinical assessments, injury prevention, and evaluating rehabilitation by monitoring recovery and improvement with therapy in both sports and pathological populations [1,2]. The use of force plates or instrumented treadmills is widely considered as the gold standard and the most direct method for measuring external loading during locomotion [3,4]. These technologies enable the precise recording of three-dimensional forces and moments, facilitating not only observational analysis but also advanced model-based methods using musculoskeletal models [5,6]. However, force plates tend to be bulky, expensive pieces of equipment that are typically embedded on a permanent or semi-permanent basis in laboratory or clinical settings. As such these environments do not necessarily reflect real-life conditions, and the conclusions drawn based on them are consequently limited [7]. Whilst useful in research, data collection in this context also places a burden on the clinicians and patients in terms of the time required to collect and process data. Therefore, in most clinical environments, direct observational methods (e.g., videos) in combination with qualitative assessments are still used due to their convenience and low cost [8]. The main limitation of these methods is their subjective nature and reliance on the experience of clinicians; they do not generate objective data that can be used to quantify deficits or improvements.

More recently, wearable sensor systems have opened up the possibility of moving gait analysis out of the lab [9], as well as finding applications in the control of exoskeletons [10]. Portable in-shoe sensors are one example, providing the user with information on loading during locomotion through one or several regions of the foot. Several studies have developed insole-type sensors using force sensitive resistor (FSR)-based technology [11], capacitive sensors [12], or piezoresistive sensors [13]. In order to be unobtrusive, these sensors have to distinguish themselves by being thin, flexible, and lightweight [14] while being subjected to large forces up to 1.5–2 times a person’s body weight. Typically, these insole sensors are designed to map the overall pressure distribution between the foot and the shoe, rather than estimate the GRF or the COP. Additionally, commercial insole technology remains expensive (e.g., EUR 7500 to EUR 15,000 for comprehensive packages [15]), limiting their widespread usage and application to daily-life activities and extended recording periods outside of laboratory settings. Ongoing research on cheaper alternatives has recognized this gap in the available technological solutions [8].

In this study, we present a low-cost 3D-printed insole capable of measuring the GRF and the anterior–posterior COP during walking. Our approach is novel in its use of pneumatic chambers and pressure sensors to estimate the forces applied by the person on the insole. The insoles are created from easily accessible, soft materials using 3D printing technology. The motivation to use these technologies comes from the ease of fabrication, which facilitates the rapid production of these insoles in different sizes and allows for personalization. In addition, the associated low cost allows these sensors to be widely used in real-world settings and over longer periods of time. Previous research on the development of 3D-printed soft pneumatic sensors also identified the versatility and effectiveness of these sensors [16,17], which compare favourably to much more expensive sensor technologies. In the following sections, we describe the design and development of the insoles, followed by the sensor characterization and the validation experiments.

## 2. Methods

In the following section, we outline the methodology and design process of the instrumented shoe with two embedded pressure-sensing chambers (PSCs) (Section 2.1), the fabrication of the PSCs (Section 2.2), the data acquisition of the PSCs, and the integration of the shoe with the chambers (Section 2.3).

### 2.1. Insole Design

**Figure 1:**
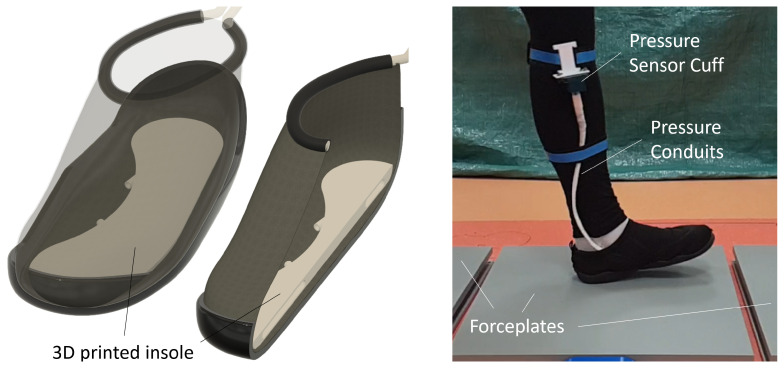
(**Left**) Illustration of the 3D-printed insole placed inside a shoe. (**Right**) Participant walking on the force plates while wearing the shoe with insole. Conduits connected the pneumatic chambers to pressure sensors, with data recorded on a portable DAQ.

**Figure 2:**
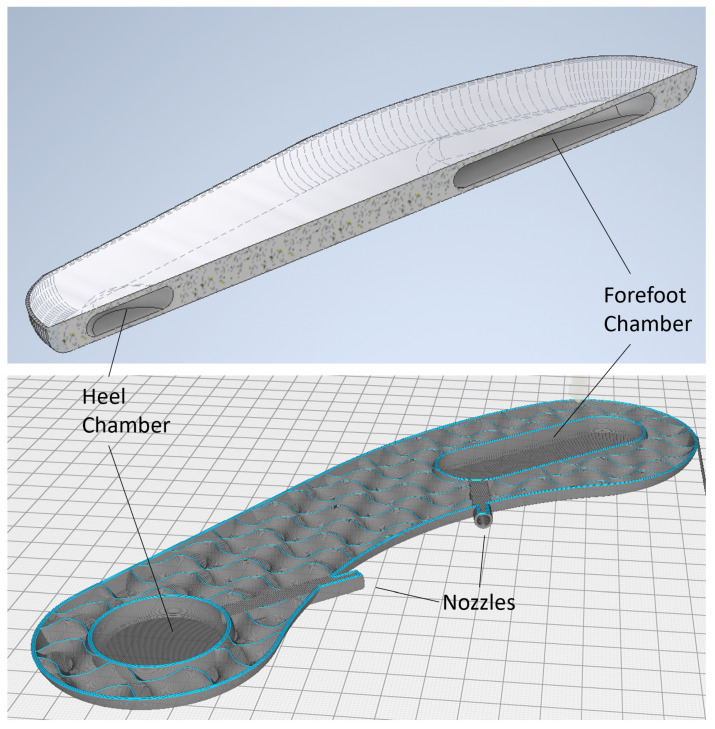
(**Top**) Sectional view shows the heel and forefoot chambers embedded within the insole. (**Bottom**) Final design of the 3D-printed insole showing the inner geometry of the chambers, the nozzles, as well as the section around the chambers. Note that the limited infill pattern is used to enable flexing of the insole during walking while providing structural support.

The overall contact between the foot and the ground was captured using two PSCs—henceforth referred to as the heel and forefoot chambers (Figure 1 and Figure 2)—embedded within the insole. The motivation for the placement, geometry, and size of the chambers came from the typical pattern of foot pressure distribution during gait. The literature has shown that, during static standing, the maximal pressure is concentrated under the heel. When transitioning to walking, the load is transferred to the forefoot as the heel leaves the ground, and thus the toes take up the load [18]. In contrast, the role of the midfoot is less significant in transferring weight from the hindfoot to the forefoot [19]. Of particular relevance to our design, a high-pressure distribution has been observed in the medial and lateral calcaneus—from the first to fifth metatarsals—and the hallux [20].

Previous research on PSCs has also shown that smooth, uniform shapes (e.g., spheres, ovals) perform more reliably than ones with sharp edges [16,17]. Based on these considerations, we designed the heel chamber with a circular profile such that it would cover the entire heel of the foot (Figure 2). The placement of the chamber was sufficiently posterior on the foot so that the chamber compression would occur at heel strike during walking. The forefoot chamber was elongated along the anterior–posterior axis of the foot, starting just behind the ball of the foot and extending past the metatarsals.

The motivation for this shape and placement was based on the results from pilot experiments and followed an iterative design process summarized in the following. An initial simple design, from the fabrication point of view, consisted of two identical circular chambers glued to a commercial flat insole and placed directly under the ball and heel of the foot. During the tests, this design proved unsuitable, as the lack of structure around the chambers was uncomfortable for the user and resulted in excessive sensor movement during walking. To mitigate the sensor movement, our next iteration consisted of 3D printing the entire insole with the circular chambers embedded into the insole. While this design prevented chamber movement and provided a more comfortable experience, we noted that the chambers were unable to capture the GRF and COP characteristics in the mid-foot region. The current forefoot chamber design (Figure 2) was therefore developed such that the latter half of the step until toe-off could be recorded.

Conduits and nozzles with a diameter of 4 mm were also embedded in the insole so that the pressure changes within the PSCs could be recorded by an external pressure sensor using the appropriate tubing. The insole with PSCs, conduits, and nozzles had a total thickness of 9 mm and a PSC wall thickness of 1 mm (i.e., the air pocket within each PSC was 7 mm thick). The insole shape and size were designed to fit the participant and the shoe used in the pilot trials (described further in Section 2.4. For the present study, we only designed and tested the insole for the left foot; however, the designs are generalizable to both feet. The 3D model of the insole in a stereolithography format is available in Supplementary Data S1.

### 2.2. Material Choice and Fabrication

Commonly used soft materials for 3D printing include thermoplastic polyurethane (TPU) and thermoplastic elastomer (TPE), both of which are available in a range of shore hardness values. TPU is a soft material with high flexibility and endurance, and it is ideal for applications requiring frequent material flexing [16,17]. In the present study, we used eSun TPU with a shore hardness value of 95A, coupled with a fused deposition modelling (FDM) printer Ender-3-S1 (Shenzhen Creality 3D Technology, Shenzhen, China). One of the challenges in using TPU for this application was that the PSC would need to be weight bearing and completely airtight. As shown in previous work, TPU-based PSCs were sensitive to changes in the printing speed. Printing speeds in the range of 7 to 15 mm/s for the PSC walls generally resulted in high quality and a sealed finish. We also found that the removal of supports (introduced during printing as stabilizers) resulted in weak spots on the PSC skin that eventually leaked. Several printing settings were iteratively modified to eliminate these defects. The Outer Wall Inset was set at 0.05 mm (Supplementary Data S2), which fused part of the outer wall line into the previous line to filter out the micro gap between them. The infill multiplier was set at 1.1, along with a 105% flow rate to increase the adhesion between every print line. Because TPU is a highly elastic material, repeatedly moving the filament flow in and out of the nozzle may create residuals that become stuck at the extruder. Therefore, the retraction distance and speed were minimized to prevent under-extrusion or clogging. The detailed settings used for printing are listed in Supplementary Data S2.

The two PSC chambers were connected to two 60 Psi pressure sensors (Honeywell, Charlotte, NC, USA) via a tubing fed through the midfoot area. The sensors themselves were mounted on a Vero board and secured to the shank with a custom-built lightweight housing (Figure 1). Analog pressure signals (in volts) were recorded at 10 kHz for the bandwidth estimation trials (Section 2.3) and 1 kHz for the characterization trials (Section 2.4), using a data acquisition hub (DAQ), DAQ-USB6009 (National Instruments, Austin, TX, USA).

### 2.3. Measurement Range and Bandwidth

In order to evaluate the efficacy of the 3D-printed insole and to test if it would be fit for our intended purpose, we conducted two tests that measured the following: (1) the overall working range and (2) the maximum measurement bandwidth. The first test was to ensure that the insole would be capable of withstanding the repeated loading of an individual’s full body weight, including dynamic loading during walking, without rupturing or suffering structural failure. The second test was to establish the sensitivity of the PSC chambers to ensure that the insole could sufficiently capture the frequency of signals typically associated with gait. The performance of the insole within the expected working range was measured by having a participant wear the shoe with the insole and stand on the force plates (Accugait, AMTI, Watertown, MA, USA), with a double-legged and a single-legged stance. The dynamic loading of the insole was simulated by shifting the body weight in the anterior–posterior direction in both stances. We noted that the insole chambers remained airtight and intact under dynamic loads of up to 900 N, as measured by the force plates, indicating that the material and design choice were sufficient for the intended application.

Bandwidth was estimated as per the methodology presented by Alici et al. [21] and Bottin-Noonan et al. [17]. Briefly, the method consisted of applying a mechanical impulse to each chamber separately and then measuring the response of the pressure signals to this impulse. By extracting the peak magnitude and time of each successive peak of the pressure signal (after impulse application), the bandwidth could then be estimated by comparison to the response of a damped harmonic system [21]. These tests were repeated 10 times for each PSC, resulting in an estimated maximum bandwidth of 247.85 ± 4.77 Hz for the heel chamber and 183.40 ± 4.34 Hz for the forefoot chamber. We noted that both PSC bandwidths were more than sufficient to measure the frequency of the signals associated with gait (typically less than 10 Hz [22]). Further details of the tests for the measurement range and bandwidth are available in Supplementary Data S3.

### 2.4. Characterization Experiments

**Figure 3:**
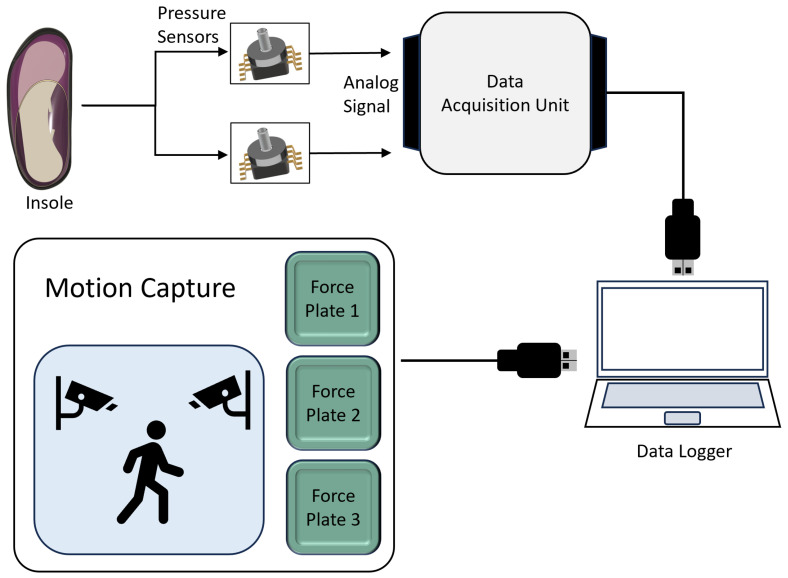
Schematic outlines the communication and data acquisition for the insole and characterization experiments. Analog pressure signals were recorded from the heel and forefoot chambers by a DAQ, onto a data logger. For the characterization experiments, additional data were recorded using a motion capture system.

We designed characterization experiments in order to map the PSC pressure signals to the GRF, as well as the anterior–posterior COP during walking. Figure 3 shows the scheme used for this step. One male participant (23 years; 79 kg; 183 cm) took part in this experiment. In addition to the instrumented shoe/insole described earlier, we utilized a optical motion capture system consisting of 6 Optitrack Prime x13 cameras (NaturalPoint, Corvallis, OR, USA) recording data at 100 Hz via the software Motive 3.0. The cameras recorded the position of reflective markers attached to a participant’s clothes/body during walking. For the purpose of this study, we only recorded the lower body motion with 20 markers on the pelvis and both legs, using Optitrack’s default “Baseline Lower” skeleton marker set [23]. The participant walked on three force plates that had been laid out in the motion capture recording space, such that a full gait cycle consisting of left–right–left steps could be recorded. The force plates recorded the orthogonal components of the forces and moments applied at 500 Hz, which were then synchronized with the motion data. Synchronization between the DAQ recording the insole pressure signals and the motion capture data was achieved by using a trigger signal sent at the start of each recording. The participant was asked to walk while stepping on the force plates at their subjective normal, slow, and fast walking speeds. Each recording trial lasted 1 min, with the participant turning around after stepping off the last force plate and repeating the steps in the opposite direction. Each trial was repeated 3 times. Overall, 258 steps with the left foot were recorded, of which 23 were excluded from further processing due to issues such as loss of signal or the participant stepping across two force plates simultaneously.

**Figure 4:**
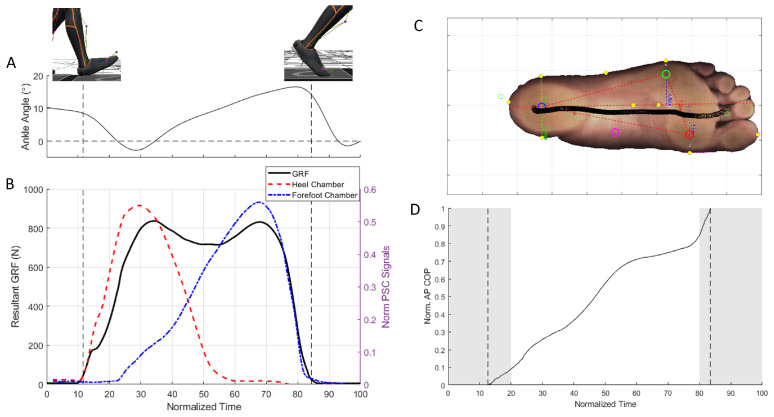
(**A**) Ankle dorsiflexion–plantarflexion angle during a representative step. (**B**) The resultant GRFs recorded by the force plates (black solid line) during a step were used detect heel strike and toe-off events (vertical dashed lines). Also shown are the recorded heel (red dashed line) and forefoot (blue dot-dashed line) pressures. (**C**) Progression of the COP during a step superimposed. (**D**) The anterior–posterior position of the COP was normalised. Vertical lines indicate heel strike and toe-off events. Shaded regions indicate parts of the step that were excluded in the model identification process.

All data were low-pass filtered at 10 Hz using a zero-lag second-order Butterworth filter. Individual steps were segmented by first identifying the points where the resultant GRF $F_{r}$ crossed a threshold value. $F_{r}$ was calculated at each recorded time step as follows:

(1) $$F_{r}=\sqrt{f_{x}^{2}+f_{y}^{2}+f_{z}^{2}}$$

where $f_{x}$, $f_{y}$, and $f_{z}$ were the orthogonal force components recorded by the force plates. These points indicated the approximate heel strike and toe-off events, and we segmented the data from 0.25 s before heel strike to 0.25 s after toe-off. This additional data was included to capture the full behavior of the PSCs going from before heel strike to after toe-off. Figure 4A illustrates the segmented GRF data, as well as the corresponding signals from the two PSCs, for one step. The positions of the center of pressure during stepping were calculated by the force plates in the laboratory frame of reference. These positions were normalized along the posterior–anterior axis of the foot, using the recorded positions of heel and toe markers (Figure 4B). For each segmented step, we extracted the resultant GRF, PSC signals, and normalized tthe posterior–anterior COP position for further processing.

#### 2.4.1. GRF Model Parameter Identification

To estimate the GRF from the PSC signals, we setup an optimization problem using the calculated $F_{r}$ as the reference. The optimization objective was to minimize the difference between the reference and the estimated GRF $F_{est}$, which was calculated as a weighted combination of the PSC signals and their rates of change. By weighting the PSC signals separately (with weights $w_{1},w_{2}$), we could account for a different contribution from the heel and forefoot chambers. The motivation to add the rate of change of these signals (captured with the weights $w_{3},w_{4}$) was based on a previous study, where such pneumatic chambers have proven to be sensitive to the rate of loading/unloading [17]. The objective function to be minimized is as follows:

(2) $$\begin{array}{ccc} & & min.\sum_{i=1}^{N}\sum_{j=0}^{t_{i}}{\left[{\left({\bar{F}}_{r}\right)}_{j}-{\left(F_{est}\right)}_{j}\right]}^{2} \end{array}$$

(3) $$\begin{array}{ccc} & & \mathrm{with},{\left(F_{est}\right)}_{j}=w_{1}*{\left({\bar{p}}_{h}\right)}_{j}+w_{2}*{\left({\bar{p}}_{f}\right)}_{j}+w_{3}*{\left(\Delta p_{h}\right)}_{j}+w_{4}*{\left(\Delta p_{h}\right)}_{j} \end{array}$$

where i=1 to *N* are the number of steps used, with each step consisting of discrete time epochs from j=0 to $t_{i}$s. ${\bar{F}}_{r}$ denotes the resultant GRF (Equation (1)), normalized by the participant’s body weight. ${\bar{p}}_{h}$ and $\Delta p_{h}$ denote the normalized heel PSC signal and its rate of change, respectively. Similarly, ${\bar{p}}_{f}$ and $\Delta p_{f}$ denote the normalized forefoot PSC signal and its rate of change, respectively. The PSC signals were normalized using their maximum values recorded over all the walking trials. The weights $w_{1}-w_{4}$ were the optimization variables to be found, subject to the following bounds:

(4) $$\begin{array}{c} 0.5\leq w_{1},w_{2}\leq 3.5 \end{array}$$

(5) $$\begin{array}{c} -30\leq w_{3},w_{4}\leq 30 \end{array}$$

with the initial values $\left(w_{1},w_{2},w_{3},w_{4}\right)=\left(1,1,0,0\right)$. The optimization bounds were chosen in the preliminary analysis such that the weight combinations would cover the variation of GRF shapes observed across walking trials. The optimization problem was solved using the interior-point algorithm within MATLAB 2024 (Mathworks, Natick, MA, USA).

#### 2.4.2. COP Model Parameter Identification

Similar to the setup of the GRF model, the normalized anterior–posterior position of the COP during stepping was calculated from the PSC signals by solving an optimization problem that identified the COP model parameters. The reference for this problem was the normalized anterior–posterior position of the COP, $\bar{p}$, as recorded by the motion capture setup. The estimated position $p_{est}$ was calculated as a weighted combination of the PSC signals and their rates of change, with a similar motivation to use these components as that outlined for GRF identification. The objective function to be minimized is as follows:

(6) $$\begin{array}{ccc} & & min.\sum_{i=1}^{N}\sum_{\bar{j}=20\%}^{80\%}{\left[{\bar{p}}_{\bar{j}}-{\left(p_{est}\right)}_{\bar{j}}\right]}^{2} \end{array}$$

(7) $$\begin{array}{ccc} & & \mathrm{with},{\left(p_{est}\right)}_{\bar{j}}=\frac{q_{1}*{\left({\bar{p}}_{h}\right)}_{\bar{j}}+q_{2}*{\left({\bar{p}}_{f}\right)}_{\bar{j}}+q_{3}*{\left(\Delta p_{h}\right)}_{\bar{j}}+q_{4}*{\left(\Delta p_{h}\right)}_{\bar{j}}}{{\left({\bar{p}}_{h}\right)}_{\bar{j}}+{\left({\bar{p}}_{f}\right)}_{\bar{j}}} \end{array}$$

where i=1 to *N* were the number of steps used, with each step consisting of discrete time epochs on a normalized scale $\bar{j}=20\%$ to 80%. The model was restricted to this time scale due to results from the pilot analysis indicating an inaccurate estimation of COP in the initial (0–20%) and final (80–100%) of the step. This was most likely due to the low pressure signals from the heel and forefoot chambers during these phases of the step, with small variations resulting in large estimation errors in Equation (7). The weights $q_{1}-q_{4}$ were the optimization variables to be found, subject to the following bounds:

(8) $$\begin{array}{c} -2.5\leq q_{1},q_{2}\leq 2.5 \end{array}$$

(9) $$\begin{array}{c} -30\leq q_{3},q_{4}\leq 30 \end{array}$$

with the initial values $\left(q_{1},q_{2},q_{3},q_{4}\right)=\left(1,1,0,0\right)$. Similar to the GRF optimization, the weight bounds were chosen such that the range would cover the variation (across speeds) observed in preliminary analysis. The optimization problem was solved using the interior-point algorithm within MATLAB 2024.

#### 2.4.3. Validation and Comparison

For each of the walking speed conditions, we used the first two trials as training data for the GRF and COP models, and the third trial for model validation purposes; the validation trials were not used to train the model. Model validation was evaluated by using the identified weights $w_{1}-w_{4}$ and $q_{1}-q_{4}$ and Equations (3) and (7) to calculate the estimated GRF and COP. We then computed the root mean square error (RMSE) of this estimate with respect to the recorded GRF and COP.

We also evaluated the performance of our insole by comparing our GRF results with those from commercially available insoles based on capacitive sensor technology (Loadsol-ap, Novel, Munich, Germany). The Loadsol insoles were inserted into the same shoe as that used in the characterization experiment. Note that we removed our custom-built insole for these trials to avoid doubling up the insole height or having one insole influence the other. For these trials, we recorded a total of 22 steps at the participant’s subjective normal walking speed. The participant walked over the same force plate setup described in Section 2.4, and the resultant force plate GRF was compared to the Loadsol measurement.

## 3. Results

**Figure 5:**
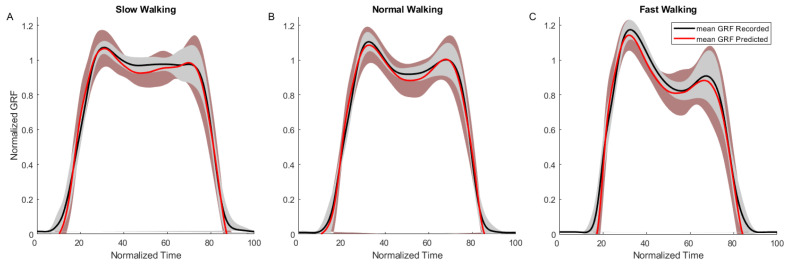
Results from the GRF model fitting for slow (**A**), normal (**B**) and fast (**C**) walking trials. Shown are the mean recorded GRF curves (black solid lines) and model prediction (red solid lines). Shaded regions indicate standard deviation over the mean across trials.

Based on the subjective choice of walking speed, the participant walked at 1.1±0.1 m/s, 1.2±0.1 m/s, and 1.4±0.1 m/s for the slow, normal, and fast walking conditions, respectively. We observed that the maximum normalized resultant GRF, $F_{r}$, increased slightly with increased walking speed, ranging from 1.07 for slow walking to 1.17 for fast walking (black lines in Figure 5A–C). There was a notable change in the profile of the GRF over time, with a more pronounced dip in the mid-stance phase for faster walking speed.

Parameter identification of the GRF model detailed in Section 2.4.1 was first conducted separately for the slow, normal, and fast walking conditions (Figure 5A–C), with the resulting weights $w_{1}-w_{4}$ listed in Table 1. RMSE between the recorded GRF and that predicted by the models were 7.2%, 6.3%, and 9.4% for slow, normal, and fast walking, respectively. We observed that, other than $w_{3}$, corresponding to the term $\Delta p_{h}$ in Equation (3), the other weights did not change appreciably across the walking speeds. Consequently, the parameter identification was rerun with all the walking speeds pooled together, as well as with the final weights listed in Table 1 and an overall RMSE of 7.7%. The identified model was used to predict GRF in the validation trials (pooled across all walking conditions), and resulted in a RMSE of 8.3%. Comparison of the GRF recorded using the force plate to that estimated by the commercial insole (outlined in Section 2.4.3) resulted in an overall RMSE of 7.0%.

**Figure 6:**
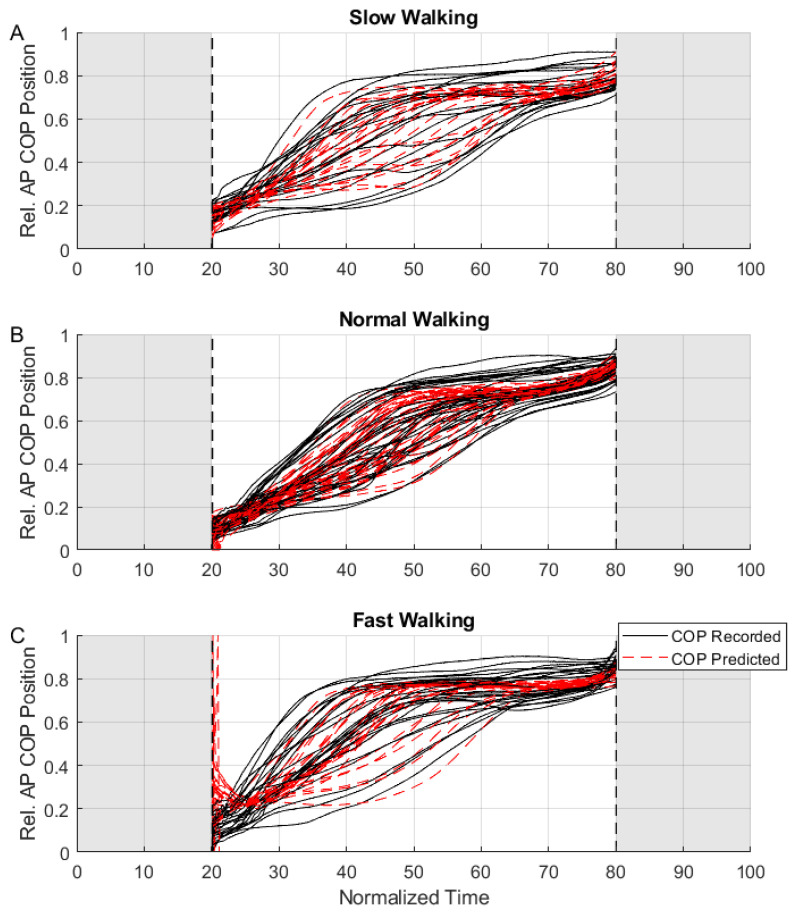
Results from the COP model fitting for slow (**A**), normal (**B**), and fast (**C**) walking trials. Shown are the recorded relative anterior–posterior COP positions (black solid lines) and model prediction (red dashed lines). Shaded regions indicate portions of the step that were excluded from the model fitting process.

Similar to the GRF processing method, the COP models were first identified separately for each walking condition. The RMSE values between the recorded and predicted COPs were 8.3%, 4.8%, and 10.3% for slow, normal, and fast walking, respectively. Pooling the walking conditions together, the overall RMSE was 9.7%, with the comparison to the validation trials resulting in an RMSE of 8.7%. Table 1 lists the identified COP model parameters. We observed that the anterior–posterior COP position varied to a larger extent for the slow walking trials (Figure 6A) and fast walking trials (Figure 6C), compared to the normal walking (Figure 6B).

The data presented here are available in Supplementary Data S4.

## 4. Discussion

The PSC-insole fabricated and tested in this study proved to be fit for the intended purpose, providing fair estimates of both the net GRF and the anterior–posterior COP position during walking. Notably, the accuracy of our system was comparable to those observed in the other off-the-shelf solutions that tend to be a lot more expensive and complex (see for example [24]). Excluding the costs of the 3D printer and the DAQ, the total cost of the PSC-insole was estimated to be about AUD 150, of which most was for the pressure sensors. Instrumented insoles in the literature have typically used up to a dozen embedded sensors [25,26]. In contrast, we chose to only use two chambers and the same number of associated pressure sensors. This design simplified the production process of the insole, making it possible to fabricate with straightforward 3D printing technology instead of requiring specialised and expensive equipment. By constraining ourselves to standard FDM printing using inexpensive printers and easily available soft materials, we also ensured that these insoles could be replicated outside of our laboratory setup.

Despite the low number of sensors, our results indicate that, with careful design of the chamber shapes and their placement, it was possible to record the overall interaction between the foot and the ground from heel strike to toe-off. Additionally, the combination of materials and shapes resulted in system bandwidths well in excess of what would be required for recording the dynamics associated with gait. This opens up the possibility of using these insoles in other applications with more dynamic movements such as running [27] and sports biomechanics [28]. However, it is important to note from the earlier research on PSC-based applications that the effects of hysteresis and non-linearity due to material softness become more pronounced with higher dynamics [16,17,29]. In this context, we note that the pattern of recorded GRF changed between slow, normal, and fast walking (Figure 5A–C). The variation (across speeds) in the identified model parameters likely reflects this change in the GRF pattern, resulting in different sets of optimal parameters. It is therefore likely that, in order to have model continuity across speeds and to predict highly dynamic movements, we would require appropriate non-linear model fitting techniques and a different treatment of the data than that used in this study. Contrary to what may be expected of pneumatic chambers made of soft materials, the PSC-insole remained airtight and intact throughout the experiments, withstanding forces up to 1.2 times the participant’s body weight and repeated usage. This is particularly remarkable considering that the thinnest parts of the insole had wall thicknesses of only 2 mm (top of insole to chamber) and 1 mm (chamber to bottom of insole).

The overall GRF error of 8.3% (from validation trials) was comparable to that of the commercial system used in this study (Loadsol) and is typical of portable sensors used for measuring daily-life conditions [30]. For context, for a person weighing 80 kg, this would mean a force error of 65 N when using the PSC-insole. Interestingly, the error was higher for slow and fast walking, indicating that, despite the high bandwidth capability of the PSC, the varying dynamics at above and below normal walking speeds influenced the behavior of the PSC. This is not an uncommon finding, especially for faster walking speeds, as larger and quicker dynamic forces would be more challenging to record. Nevertheless, even at the fast walking speeds, the overall RMSE was still below 10%.

Identification of the anterior–posterior COP position produced mixed results. From the data analysis, we noted that the position estimation did not work for parts of the step cycle where the heel and forefoot chambers were lightly loaded (essentially around the heel strike and toe-off events). This can be observed in Figure 6C around the 20% mark, where the combination of faster walking dynamics and low pressure on the heel chamber resulted in a large estimation error. Following this observation, we constrained our model estimation from 20 to 80% of the step and noted that, for this section of the step cycle, the COP position estimation had an overall error of 8.7% (from the validation trials). For context, for a person with an overall foot length of 30 cm, this would result in an anterior–posterior COP position error of 2.6 cm. We also noted that the slow and fast walking conditions resulted in a greater spread in the COP (comparing the vertical spread of the black lines in Figure 6A,C to the spread in Figure 6B). when compared to normal walking. Consequently, the COP model prediction capability was poorer under these conditions. It is relatively uncommon for portable insoles to provide COP estimates; however, our results are comparable to those from the literature [31,32]. We believe that the level of accuracy achievable by our sensors may be sufficient for applications such as studying balance [33] and knee osteoarthritis [34], where a qualitative assessment of the anterior–posterior COP in real-world settings would more relevant than a precise one in laboratory settings. However, for applications where precise COP measurements around the mid-foot area are required, further chambers (and consequently sensors) in the mid-foot region would be prudent.

We acknowledge the following limitations associated with this work and some mitigating solutions. First, we could only estimate the total magnitude of force applied by the person and not the orthogonal force components nor moments that one would typically record with force plates. This was primarily due to the limited space available under the foot and as a reasonable trade-off between sensor complexity and cost. While the force magnitude can be a useful metric, it can overlook certain clinically relevant gait characteristics such as excessive shear forces at the foot during walking (e.g., in shuffling gait). It should, however, be noted that most portable insoles do not provide independent force or moment components. There has been some research on using soft sensors to dissociate normal and shear forces [35], which could be interesting for future research. Second, our pressure measurements were recorded off the shoe, on a cuff strapped to the shank. The conduits and especially the nozzles connecting the conduits to the PSC-insole were a weak point in the system and would likely fail after extended usage. The shank cuff was also a cumbersome component that would not be suitable for more widespread application. In the current work, we aimed to improve this design by embedding low-profile pressure sensors directly into the chambers during the 3D printing process (similar to the approach used by [26]. By eliminating the need for pressure conduits, we could then also move the signal processing and recording setup into the shoe. Finally, in this pilot work, we based our results on the data from one participant with flat overground walking. To demonstrate the viability of this technology for wider use, we aim to repeat these tests for a larger cohort and focus on evaluating the capability of the PSC-insole when walking over uneven ground, as expected in daily-life conditions.

Our work provides an interesting and novel application of 3D printing with soft materials. To the best of our knowledge, no such insole exists for measuring both the GRF and the anterior–posterior COP position. Moreover, this design was able to perform admirably compared to the much more expensive off-the-shelf solutions. By using easily accessible methods and technology, such 3D-printed insoles could be one way to make it easier to record gait parameters outside laboratories and in more widespread settings.

## Figures and Tables

**Table 1:** Identified model parameters.

		Slow	Normal	Fast	Combined
GRF	$w_{1}$	1.5746	1.4319	1.67	1.529
	$w_{2}$	1.9876	1.906	2.0363	1.9663
	$w_{3}$	−1.1669	−3.3699	4.0188	0.02
	$w_{4}$	24.8631	24.232	25.1783	25.3092
COP	$q_{1}$	0.2407	0.199	0.257	0.2193
	$q_{2}$	0.7317	0.7528	0.7573	0.7585
	$q_{3}$	−4.1693	−3.2918	1.4174	1.5132
	$q_{4}$	−5.5972	−5.2196	−1.8396	−2.5901

## Data Availability

The original contributions presented in the study are included in the article/Supplementary Materials, and further inquiries can be directed to the corresponding author.

## Supplementary Materials

The following supporting information can be downloaded at: https://www.mdpi.com/article/10.3390/s25082524/s1. S1: PSC Insole STL; S2: 3D printing parameters; S3: PSC-Insole Range and Bandwidth Tests; S4: Experimental dataset containing training and validation data.

## Abbreviations

The following abbreviations are used in this manuscript:

GRF	Ground Reaction Force
COP	Center of Pressure
PSC	Pneumatic Sensing Chamber
TPU	Thermoplastic Polyurethane
FDM	Fused Deposition Modelling
DAQ	Data Acquisition Hub
RMSE	Root Mean Square Error
