# Supplementary material for: 3D-Printed Insole for Measuring Ground Reaction Force and Center of Pressure During Walking
Source: Sensors (Basel). 2025 Apr 17;25(8):2524. doi: 10.3390/s25082524 (PMC12031006; doi:10.3390/s25082524)
Supplement: Supplementary file 1 [file sensors-25-02524-s001.zip › S2_3D_Printing_Parameters.pdf]

Supplementary Data to

3D-Printed Insole for Measuring Ground Reaction Force and Center of Pressure During Walking

Le Tung Vu, Joel Bottin-Noonan, Lucy Armitage, Gursel Alici and Manish Sreenivasa *

School of Mechanical, Materials, Mechatronic and Biomedical Engineering, Faculty of Engineering andInformation Sciences, University of Wollongong, Wollongong, NSW 2522, Australia

**Correspondence: manishs@uow.edu.au; Tel.: +61-242981332*

Table S1:

Parameter	Value	Unit
Quality		
Layer Height	0.12	mm
Line Width	0.4	mm
Shell		
Top Layers	11	layers
Bottom Layers	11	layers
Outer Wall Inset	0.03	mm
Infill		
Infill Density	5	%
Infill Line Distance	8	mm
Infill Line Multiplier	1	
Infill Overlap	30	%
Material		
Printing Temperature	235	C
Build Plate Temperature	50	C
Flow	105	%
Wall Flow	105	%
Speed		
Print Speed	40	mm/s
Infill Speed	49	mm/s
Wall Speed	25	mm/s
Travel Speed	150	mm/s
Initial Layer Speed	15	mm/s
Travel		
Retraction Distance	0.8	mm
Retraction Speed	15	mm/s
Cooling		
Fan Speed	30	%
Support		
Support Overhang Angle	45	Degree
Support Pattern	Zigzag	
Support Density	10	%
