# Supplementary material for: 3D-Printed Insole for Measuring Ground Reaction Force and Center of Pressure During Walking
Source: Sensors (Basel). 2025 Apr 17;25(8):2524. doi: 10.3390/s25082524 (PMC12031006; doi:10.3390/s25082524)
Supplement: Supplementary file 1 [file sensors-25-02524-s001.zip › S3_PSC-Insole_RangeBandwidth.pdf]

Supplementary Data to

3D-Printed Insole for Measuring Ground Reaction Force and Center of Pressure During Walking

Le Tung Vu, Joel Bottin-Noonan, Lucy Armitage, Gursel Alici and Manish Sreenivasa *

School of Mechanical, Materials, Mechatronic and Biomedical Engineering, Faculty of Engineering andInformation Sciences, University of Wollongong, Wollongong, NSW 2522, Australia
**Correspondence: manishs@uow.edu.au; Tel.: +61-242981332*

Bandwidth evaluation was the means to evaluate the effectiveness of the PSC's mechanical and voltage output responses under impact force. A high bandwidth indicates high sensitivity of the mechanical sensing unit, thereby verifying the capability of the PSC to capture high-frequency signals. Previous research has proposed an impulse test to estimate the bandwidth of the mechanical system in order to recognize the frequency response [1]. This method, derived from [1], is briefly outlined here.

The logarithmic decrement δ measures the decrement of the amplitude of the response with respect to time, as,

$$\boldsymbol{\delta = \frac{1}{n} ln \frac{Q_0}{Q_n}} \qquad \text{------ (S1)}$$

We calculated the bandwidth ω_b, as follows,

$$\boldsymbol{\omega_b = \frac{2\pi}{\tau_d\sqrt{1-\zeta^2}}\left(1 - 2\zeta^2 + \sqrt{4\zeta^4 - 4\zeta^2 + \zeta}\right)^{\frac{1}{2}}} \qquad \text{------ (S2)}$$

where $\boldsymbol{\delta = \frac{2\pi\zeta}{\sqrt{1-\zeta^2}}}$

$$\text{with} \begin{cases} \boldsymbol{Q_0; Q_n = amplitude\ of\ peaks\ at\ t_0; t_n} \\ \boldsymbol{\xi = damping\ ratio\ of\ response} \\ \boldsymbol{\tau_d = period\ of\ damped\ response} \end{cases}$$

For these tests, the pressure signals from both PSCs were sampled at 10 kHz. A mechanical impulse was applied to the upper surface of each PSC while the bottom surface was secured onto a flat base. The bandwidth of each PSC was then estimated using custom-built code in MATLAB to identify signal peaks (Figure S1) and apply Equations (S1) and (S2). We repeated 10 trials for each PSC. The results showed an average bandwidth of 183.40Hz ± 4.34Hz for the forefoot chamber and 247.85Hz ± 4.77Hz for the heel chamber.

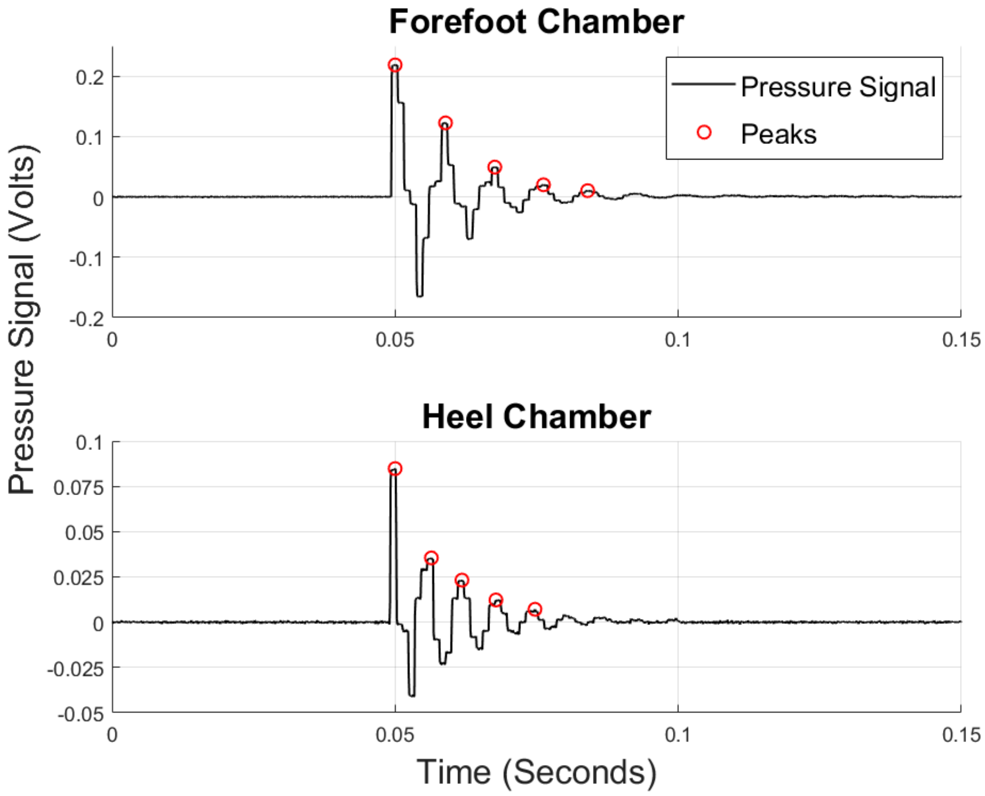

Figure S1: Bandwidth from PSC pressure signals.
